# A toolkit for measurement error correction, with a focus on nutritional epidemiology

**DOI:** 10.1002/sim.6095

**Published:** 2014-02-04

**Authors:** Ruth H Keogh, Ian R White

**Affiliations:** a Department of Medical Statistics, London School of Hygiene and Tropical MedicineLondon, U.K.; b MRC Biostatistics UnitCambridge, U.K.

**Keywords:** measurement error, regression calibration, moment reconstruction, multiple imputation, diet diary, food frequency questionnaire, nutritional epidemiology

## Abstract

Exposure measurement error is a problem in many epidemiological studies, including those using biomarkers and measures of dietary intake. Measurement error typically results in biased estimates of exposure-disease associations, the severity and nature of the bias depending on the form of the error. To correct for the effects of measurement error, information additional to the main study data is required. Ideally, this is a validation sample in which the true exposure is observed. However, in many situations, it is not feasible to observe the true exposure, but there may be available one or more repeated exposure measurements, for example, blood pressure or dietary intake recorded at two time points. The aim of this paper is to provide a toolkit for measurement error correction using repeated measurements. We bring together methods covering classical measurement error and several departures from classical error: systematic, heteroscedastic and differential error. The correction methods considered are regression calibration, which is already widely used in the classical error setting, and moment reconstruction and multiple imputation, which are newer approaches with the ability to handle differential error. We emphasize practical application of the methods in nutritional epidemiology and other fields. We primarily consider continuous exposures in the exposure-outcome model, but we also outline methods for use when continuous exposures are categorized. The methods are illustrated using the data from a study of the association between fibre intake and colorectal cancer, where fibre intake is measured using a diet diary and repeated measures are available for a subset. © 2014 The Authors.

## 1. Introduction

In epidemiology, many exposures of interest are subject to measurement error. Error arises for a range of reasons: because of error at the measurement processing stage (e.g. laboratory error), because of self-reporting, because of limitations of measurement instruments and because of fluctuations in exposure over time when the exposure of interest is ‘usual’ level and inferences are based on a single measurement. We focus on exposures measured on a continuous scale. Examples include biological exposures, such as blood pressure 1 and plasma fibrinogen 2, and measurements of dietary intake in nutritional epidemiology 3.

Exposure measurement error, which can take a variety of forms, results in bias in estimates of associations between the exposure and outcomes of interest (e.g. disease status). The severity and nature of the bias depends on the form of the measurement error. It is important to take account of the effects of measurement error when drawing inferences in epidemiological studies, and there is now a substantial literature on methods for error correction: for extensive overviews, see for example Carroll *et al*. 4 and Buonaccorsi 5. In this paper, we bring together what we consider to be the most important methods, placing an emphasis on their practical application.

To make corrections for the effects of measurement error on estimated exposure-outcome associations, additional information is required on top of the main study data in order to understand the measurement error. Ideally, this is in the form of a validation sample within the main study, in which the true exposure is observed alongside the error-prone exposure measurement. However, there are many situations in which it is not feasible to observe the true exposure. An example is when the true exposure is the underlying usual level of a quantity which varies over time, such as blood pressure or dietary intake. In this case, there may be available one or more repeated observations of the error-prone exposure measurement, for example, dietary intake recorded at two or more time points. In this paper, we focus on this situation, though we also comment on the situation where the true exposure is observed for some individuals.

The aim of this paper is to provide a toolkit for measurement error correction, which covers different types of error. Our main focus is on using continuous exposures in the exposure-outcome model, in particular where we assume a linear association, on the appropriate scale. However, we also outline methods for use when continuous exposures are categorized in the exposure-outcome model, a common practice in epidemiological analysis. We place an emphasis on measurement error correction in the context of nutritional epidemiology, where methods for error correction using repeated measures are of growing importance. However, all methods described also apply in a more general setting.

In Section 2, we describe some of the issues of measurement error which arise in nutritional epidemiology, which partially motivated this paper. In Section 3, we set the scene by outlining the exposure-outcome model of interest and by describing different models for measurement error. Alongside the most commonly assumed classical error model, we also consider systematic error, heteroscedastic error and differential error. Both univariate and multivariate exposures are considered. In Section 4, we outline measurement error correction using regression calibration (RC) under different error models. Two more recently described correction methods are then described in Section 0015: moment reconstruction (MR) 6,7 and multiple imputation (MI) 8,7. Unlike RC, these methods can accommodate differential error. Section 0018 focuses on correction for error due to misclassification when mismeasured continuous exposures are categorized. In Section 0022, the methods are illustrated using data from a study of fibre intake and colorectal cancer 9. We conclude with a discussion in Section 0026.

## 2. Measurement error in nutritional epidemiology

The exposure of interest in nutritional epidemiology is typically the long term average, or ‘usual’, daily intake of a given nutrient, food or food group. Studies rely on self-reported measures of intake, which are subject to error. It is not feasible to observe the true exposure, so studies of measurement error depend on repeated exposure measurements or comparisons with a superior measure. The main methods for obtaining self-reported measures of dietary intake are food frequency questionnaires (FFQs) or food records, which include 24-h recalls and diet diaries 3. There exist objective biological measurements of absolute intakes of a very limited number of nutrients (total energy and the nutrients protein, potassium and possibly sodium) 10–12, though their expense prohibits use in large samples.

Food frequency questionnaires provide a relatively inexpensive method of measuring dietary intake compared with food records and have been used as the main dietary assessment instrument in most large prospective studies in nutritional epidemiology to date (e.g. 13,14). However, validation studies comparing biological and self-reported measurements have found that food record measurements are more highly correlated with both the biomarker and true intake than FFQs 15–17. Food records therefore currently arguably provide the best available self-reported dietary assessment instrument. Food record measurements have typically been assumed to be subject only to random error. However, there is evidence from comparisons with objective biomarkers that food record measurements are subject to person specific errors and error that depends on the true level of exposure 15,17,18,16.

In this paper, we provide a set of measurement error correction methods which can be applied in studies where the main exposure measurement is error-prone, but where repeated exposure measurements are available in a subset of the study population. This work was motivated by the growing use of food records in epidemiological studies, where repeated measures are often available. In some recent studies, food records have been used as the main instrument in case–control studies nested within cohorts 19,20. One example, the UK Dietary Cohort Consortium, is a collaboration between several UK cohorts, which have collected diet diaries for all or a subset of participants 9,21–27. Repeated measures were available in a subset. Web-based food records are growing in use and make it feasible to obtain food record data, including repeated measures, for large sample populations. One example is the automated self-administered 24-h recall (ASA24) developed by the National Cancer Institute, US National Institutes of Health (http://riskfactor.cancer.gov/tools/instruments/asa24/).

The methods described apply equally well for FFQs, where repeated measures are available. However, some assumptions will be less plausible for FFQs. For example, some investigators are prepared to assume that food record measurements are subject only to random error, whereas this would not be the case for FFQs. One particular contribution of this paper is to outline the use of sensitivity analyses to investigate the potential impact of non-random errors, which is important in this dietary setting. The methods we describe are not in general appropriate for use with measurements of mixed types, for example, an FFQ measurement plus a food record measurement. There is quite a large literature on methods for error correction using measurements of mixed types in nutritional epidemiology (see, for example, 28,29 for summaries).

## 3. Setting the scene: notation and models for error

We let **X**_*i*_ denote a vector of true but unobserved continuous exposures for individual *i*. The vector of mismeasured exposures for individual *i* is denoted **W**_*i*1_, and we assume that this is observed for all individuals in the study population. As noted earlier, additional observed exposure measurements are required to perform corrections for the effects of measurement error on exposure-outcome associations. A vector of repeated observed exposure measurements is denoted **W**_*i*2_. The error in **W**_*i*2_ will be assumed to have the same distribution as that in **W**_*i*1_. An alternative would be for external information to be available about the form of the error, for example, from a previous study. We do not consider that situation here. In some, but not all, of the correction methods which we will describe, assumptions are required concerning the distribution of **X**_*i*_. In Sections 4–6, we note the assumptions used in each of the correction methods.

### 3.1. The exposure-outcome model

The focus here is on a binary univariate outcome *Y*_*i*_, for example, disease status, and on the use of a logistic regression to investigate the exposure-outcome association. The focus is on the logistic model because of its wide use in epidemiology. However, we will also comment in places on other analysis models, specifically linear regression and proportional hazards regression in survival analysis. Adjustment variables measured without error (categorical or continuous) are summarized in a vector **Z**_*i*_. We assume the following linear association between true exposure and the outcome on the logistic scale:

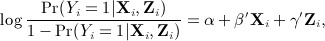
1 where *β* is a vector of log odds ratios (ORs), which are assumed to be the parameters of primary interest. Models involving non-linear functions of **X**_*i*_ are discussed in a later section. The naive approach to estimating *β* is to use **W**_*i*1_ in place of **X**_*i*_, giving the model

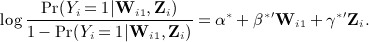
2 However, if the measurements **W**_*i*1_ are subject to error, 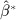 will be a biased estimator for *β*. The other parameters estimated under model 2 will also be biased but are of less interest.

### 3.2. Classical measurement error

First, consider the univariate situation, that is, the situation in which there is one error-prone exposure of interest. The classical measurement error model is

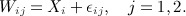
3 where the *ε*_*ij*_ are error terms with mean 0 and constant variance 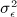, and where there is zero correlation between *ε*_*i*1_ and *ε*_*i*2_. The *ε*_*ij*_ are independent of *X*_*i*_, **Z**_*i*_ and *Y*_*i*_.

Sometimes, there may be multiple mismeasured exposures of interest. For example, in the context of nutritional epidemiology, it is common to adjust for total energy intake. We extend the aforementioned definition of classical measurement error to the multivariate situation. The vector of true exposures for individual *i* is 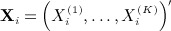, and the corresponding vector of observed exposures is 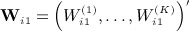. The aim, in this multivariate case, is to estimate the parameters **β** = (*β*^(1)^, … ,*β*^(*K*)^) ′  in the exposure-outcome model in 1. Assuming the classical error model for all exposures, we can write

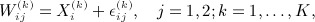
4 where all the errors 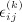 have mean 0 and are independent of each other and of **X**_*i*_, **Z**_*i*_ and *Y*_*i*_.

### 3.3. Systematic error depending on true exposure

Under the classical error model in 3, the *W*_*ij*_ are described as unbiased measures of *X*_*i*_ because the average over a large number of repeated measurements would provide an estimate of *X*_*i*_. In some cases, the classical error model may be unrealistic. For example, in nutritional epidemiology there is evidence that food record measurements are biased estimates of true intake. Specifically, studies have found evidence that food record measurements are subject to error which depends on the true exposure *X*_*i*_ and also to person specific errors 15,17,16, that is, errors which are correlated across repeated measurements within an individual. A more realistic model for error in food record measurements may therefore be, using univariate notation,

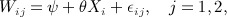
5 where *ψ* is a constant shift, *θ* ≠ 1 represents error dependent on true exposure and correlations between errors may in general be non-zero. We denote the error correlation by corr(*ε*_*i*1_,*ε*_*i*2_) = *ρ*. It is still assumed that the *ε*_*ij*_ have zero mean and constant variance and are independent of *X*_*i*_, **Z**_*i*_ and *Y*_*i*_. This model reduces to the classical measurement error model when *ψ* = 0,*θ* = 1,*ρ* = 0. The parameters {*ψ*,*θ*,*ρ*} cannot be estimated using repeated measures of *W*, and in a later section we suggest the use of sensitivity analyses to incorporate different values for these parameters into corrected estimates of the exposure-disease association.

### 3.4. Heteroscedastic error

In the error models described earlier, the error terms *ε*_*ij*_ had constant variance. An alternative error model we consider is one in which the error variability depends on *X*_*i*_, typically increasing with *X*_*i*_ so that observed values for individuals with higher true exposure are subject to greater measurement error. Under this assumption, we may express a measurement error model for *W*_*ij*_ as 3 or 5 with

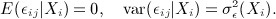
6 Note that under this model, the observed exposure measurements are still assumed to be unbiased for *X*_*i*_. Other formulations are possible, but we do not discuss them here. If there is a subset of the study population in which the true exposure *X*_*i*_ is observed, then it is possible to investigate graphically whether the variation in *W*_1_ changes with *X*. When *X*_*i*_ is not observed, it is possible to partly test for classical measurement error if a repeated measure of the exposure *W*_*i*1_ is available by plotting the standard deviation of {*W*_*i*1_,*W*_*i*2_} against the mean of {*W*_*i*1_,*W*_*i*2_}for each individual. This will indicate whether the error variance depends on the true exposure 4.

In many situations, it may be possible to apply a transformation to the original variable *W*_*ij*_ to obtain values which have the desired property of constant error variance. In the simplest case, a log transformation may be suitable. More generally, a Box–Cox transformation may be found 30.

### 3.5. Differential error

In the error models described earlier, the error was assumed independent of the outcome. This is referred to as non-differential error. The final type of measurement error we consider in this paper is differential error, in which the error depends in some way on the outcome, that is, on the binary variable *Y*_*i*_ in our situation. This type of error may occur if exposure information is obtained in a different way from cases and controls or if disease status in some way affects the error (e.g. due to recall bias). Differential error may feature as an extension to any of the error models described so far. For example, under the classical error model in 3, the variability of the errors *ε*_*ij*_ may differ for *Y*_*i*_ = 0,1. The systematic error model in 5 may be extended to allow the systematic bias to depend on a binary *Y*_*i*_:

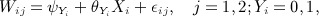
7 with the possibility again that error variances also depend on *Y* (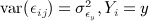), and further that error correlations may depend on *Y* ( corr(*ε*_*ij*_,*ε*_*ik*_) = *ρ*_*y*_,*Y*_*i*_ = *y* for *j* ≠ *k*). Other differential error models are possible; a simple model in which individuals with *Y*_*i*_ = 1 have a mean shift in *W*_*ij*_ relative to *Y*_*i*_ = 0 is a special case of 7 and may be written

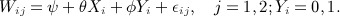
8

### 3.6. Effects of measurement error

We comment briefly here on the impact of measurement error on observed exposure-outcome associations obtained by fitting model 2.

If there is a linear relationship between a univariate exposure and the outcome, as under model 1 and similar linear and Cox regression models, then the effect of classical measurement error in a univariate exposure is to attenuate the exposure-outcome association 4,31. That is, estimates of exposure-outcome associations, such as a log OR, a regression coefficient in a linear regression or a log hazard ratio, will be biased towards the null. A further effect of classical measurement error in linear models is loss of power to detect exposure-outcome associations.

Classical error in a multivariate exposure setting can result in bias in any direction even in a linear regression model 4. Where there are non-linear terms, for example, a quadratic term, in the exposure-outcome model, classical measurement error has the effect of making the association appear more linear 32. Other types of error, such as systematic error, which depends on the true exposure; heteroscedastic error; and differential error, which depends on the outcome, may in general result in biases of any form, for example, bias either away from or towards the null 4,33.

## 4. Measurement error correction using regression calibration

The overall aim is to obtain an unbiased estimate of the parameter vector **β** in model 1, which in this case is a vector of log ORs.

The first method for measurement error correction that we describe is RC, which is probably the most commonly used approach. Under RC, **β** is estimated by using *E*(**X**_*i*_ | **W**_*i*1_,**Z**_*i*_) in place of **X**_*i*_ in model 1 4. Under a linear regression model for the exposure-outcome model, this procedure results in unbiased estimates of **β**. However, it also works approximately under non-linear regression models, including the logistic model. The approximation required for non-linear analysis models has been found to perform well under many circumstances for logistic regression 31,34–36. RC also extends approximately to proportional hazards regression 37,38.

The use of RC relies crucially on the assumption that, conditionally on **X**_*i*_, **W**_*i*1_ provides no additional information about disease risk, that is, the error in **W**_*i*1_ is non-differential. Error correction methods which can accommodate differential error are considered in a later section.

In order to perform RC, we need to find the expectation *E*(**X**_*i*_ | **W**_*i*1_,**Z**_*i*_). This is outlined in the next two sections for univariate and multivariate exposures under the assumption of a classical measurement error model and extended to systematic error and heteroscedastic error in later sections.

### 4.1. Classical measurement error: univariate exposure

In the case of a univariate exposure measured with error, *W*_*i*1_, the expectation *E*(*X*_*i*_ | *W*_*i*1_,**Z**_*i*_) required to perform RC can be found by using the linear regression relation

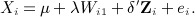
9 We refer to 9 as the RC model. It is important that any adjustment variables in the exposure-outcome model, **Z**_*i*_, are included in the RC model. By using the RC model, the expectation is given by 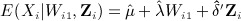, which is then used in place of *X*_*i*_ in 1 to obtain the estimate of *β*. This procedure results in an estimate of *β*, which is exactly equivalent to

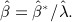
10 That is, in the univariate case, there are two ways to estimate *β* using RC. Both require fitting of the RC model 9. One is to find *E*(*X*_*i*_ | *W*_*i*1_,**Z**_*i*_) and use the expected values in the main analysis model 1; the other is to fit the naive model 2 to obtain 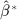 and use formula (10). This second method is popular because of its simplicity. The correction factor *λ* is referred to as the attenuation factor or regression dilution ratio (RDR); we use the latter term in the remainder of the paper.

The RC model can be fitted easily if the true exposure *X*_*i*_ is observed in a subset of individuals within the study. Here, however, we focus on a situation in which the true exposure cannot be observed. Suppose that a repeat of the univariate exposure measurement *W*_*i*1_ is available for at least a subset of the study population; *W*_*i*2_. Under the classical measurement error model 3, the parameters in the RC model 9 can be estimated by a linear regression of *W*_*i*2_ on *W*_*i*1_ and **Z**_*i*_:

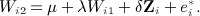
11 The right-hand sides of RC models (9) and (11) differ only by the error term, and it can be seen that 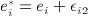, where *ε*_*i*2_ is the error in *W*_*i*2_ under the classical error model 3. No assumptions about the joint distribution of {*X*_*i*_,*W*_*ij*_,**Z**_*i*_}are required for the methods described in this section to be valid. The estimate of *λ* obtained using (11) is 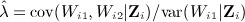.

When using RC, error in the estimation of the RDR *λ* should be carried through when estimating uncertainty in *β*. The variance of 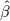 can be found using a second order Taylor approximation (the ‘delta method’), giving

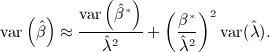
12

Bootstrapping would be an alternative way of estimating 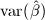. We do not go into the details of this, because the method of bootstrap sampling would depend on the study design.

In many cases, it is likely that the error in estimation of *λ* will be small relative to the error in estimation of *β*, and for this reason, sometimes *λ* is assumed to be estimated without error.

An extension to RC, called ‘efficient RC’ 39,7 has been suggested, which makes more efficient use of the measurements available for individuals in a validation study or repeated measures available for a subset. When there is a validation study within which *X* is observed, the efficient RC estimate of *β* is an inverse variance weighted average of the estimate of *β* within the validation study and the estimate of *β* using (10). We do not give the details here.

In the case of a logistic exposure-outcome model, a better approximation to (10) can be found by using a probit approximation to the logistic function 36. Under the classical error model 3, it can be found that

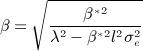
13 where 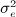 is the variance of the residuals in the RC model 9 and *l* ≈ 0.59, which stems from the probit approximation. When 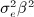 is small, the aforementioned result reduces to (10).

### 4.2. Classical measurement error: multivariate exposure

Regression calibration can also be used in the multivariate situation, where more than one exposure in the exposure-outcome model is measured with error. In this case, the vector of log OR parameters **β** is estimated by replacing each element of **X**_*i*_ by its expectation conditional on all observed measurements, that is, 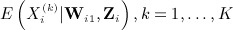. In a direct extension to the univariate procedure, the expectations can be found by assuming linear regression models

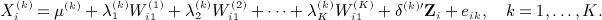
14 When **X**_*i*_ cannot be observed in a validation sample, repeated measurements are required for all dietary exposures for at least a subset of the study population, in order to estimate the parameters in the multivariate RC model (14). Under this assumption, model (14) can be fitted by linear regressions of 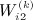 on **W**_*i*1_ and **Z**_*i*_, for each dietary exposure *k* = 1, … ,*K*.

The simplest way of performing the correction is to obtain the expectations 
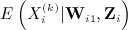
 (*k* = 1, … ,*K*) explicitly and use them in place of 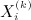 (*k* = 1, … ,*K*) in the exposure-outcome model. Alternatively, a matrix of RDRs can be obtained and applied as a correction to the observed parameter vector *β*^ *^ 33. As in the previous section, no assumptions are required here about the joint distribution of {**X**_*i*_,**W**_*ij*_,**Z**_*i*_}.

Variability in the estimates of parameter in the RC model (14) should be carried through to the corrected estimates of *β*. The delta method approach for the univariate situation, as described inSection 4.1, has been extended to the multivariate case 33. However, bootstrapping appears to be the simplest approach in the multivariate case.

### 4.3. Extension to systematic error

Suppose now that the model for measurement error is of the systematic form given in 5. In the univariate situation, we denote the correlation between the errors *ε*_*i*1_ and *ε*_*i*2_ by *ρ*. In this case, the parameters {*μ*,*λ*,*δ*}of the RC model cannot be estimated using repeated exposure measurements as in model (11). The RC model in 9 could be fitted if the true exposure were available from a validation study in a subset of the study population. Alternatively, the RC model could be fitted if an unbiased measure of the true exposure were available, by using that in place of *X*_*i*_. For example, in nutritional epidemiology, an unbiased biomarker could be used in the rare cases where they exist. Here, we assume that such measures are not available, and we propose using sensitivity analyses to investigate the effects of different values for the bias parameters {*ψ*,*θ*,*ρ*}in model 5 using repeated exposure measurements.

Under the error model in 5, we have cov(*X*_*i*_,*W*_*i*1_ | **Z**_*i*_) = *θ*var(*X*_*i*_ | **Z**_*i*_). Under the assumption that var(*W*_*i*1_ | *X*_*i*_,**Z**_*i*_) = var(*W*_*i*2_ | *X*_*i*_,**Z**_*i*_), it can be shown that

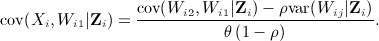
15 It follows that the RDR, parameter *λ* in the RC model 9, is estimated by

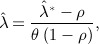
16 where 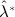 arises from the linear regression of *W*_*i*2_ on *W*_*i*1_ and **Z**_*i*_; *W*_*i*2_ = *μ*^ * ^ + *λ*^ * ^*W*_*i*1_ + *δ*^ * ′ ^**Z**_*i*_ + *η*_*i*_. The log OR of interest, *β*, can therefore be estimated as in (10). Note that specification of a value for the measurement error model intercept *ψ* is not required. Although the parameters **δ** in model 9 may not generally be of interest, they can be estimated by 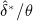. The intercept *μ* in the RC model 9 cannot be estimated without specifying *ψ* in 5, but we are not typically interested in *μ*.

The variances of the corrected estimates can be estimated using the approximation in (12), replacing *λ* by *λ*^ * ^.

The choice of suitable values for parameters {*θ*,*ρ*}for use in a sensitivity analysis will be dependent on the subject matter. In a later section, we discuss choice of suitable values in a study in nutritional epidemiology.

In the multivariate case, the systematic error model for the *k*th exposure is 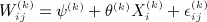, where 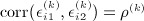. Sensitivity analyses could be used to assess the effects on results of systematic errors. However, this would require a number of parameters to be specified simultaneously, which soon becomes impractical, and we do not give the details here. It is worth noting that the values of *θ*^(*k*)^ and *ρ*^(*k*)^ in the measurement error model do not affect the estimate of the *β*^(*l*)^ (when *k* ≠ *l*).

### 4.4. Heteroscedastic error

Correction for heteroscedastic error has received considerably less attention in the literature than classical error. As noted earlier, constant error variance may be achievable after a transformation of the error-prone exposure. In the exposure-outcome model, it may be considered appropriate to use the transformed scale measurement as the main exposure, that is, to assume a linear association between the transformed exposure and outcome. In this case, RC can be applied as in the preceding text, using the transformed scale measurements. If, on the other hand, it is desirable to use the untransformed exposure in the exposure-outcome model, that is, to assume a linear association between the untransformed exposure and outcome, then difficulties arise.

We focus on the special situation in which a measurement error model with constant variance, 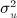, can be achieved after a log transformation. Suppose first that the error model for *W*_*ij*_ is

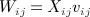
17 where *v*_*ij*_ is a multiplicative error. If *W*_*ij*_ is normally distributed on the log scale, and under an assumption that *W*_*ij*_ is unbiased measure of *X*_*i*_ on the *original* scale, it can be shown that

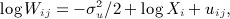
18 where *u*_*ij*_ has a normal distribution with mean 0 and constant variance 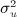 or equivalently log *v*_*ij*_ has a normal distribution with mean 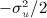 and variance 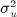. That is, the log scale exposures follow a classical error model with a constant shift. We suppose that log *W*_*ij*_ is normally distributed with mean *μ*_*W*_ and variance 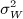 and that log *X*_*i*_ is normally distributed with mean *μ*_*X*_ and variance 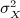, where 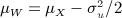 and 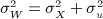. In this case, the expectation of interest for use in RC, *E*(*X*_*i*_ | *W*_*i*1_,**Z**_*i*_), can be evaluated exactly 4 and is given by

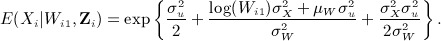
 Using *E*(*X*_*i*_ | *W*_*i*1_,**Z**_*i*_) in place of *X*_*i*_ in the exposure-outcome model is equivalent to fitting the model with 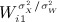 in place of *X*_*i*_ and then dividing the estimated slope parameter by 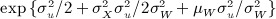. Note that these results rely on the assumption that log *W*_*ij*_ and log *X*_*i*_ are jointly normally distributed, whereas the use of RC under the classical error model in 3 does not require assumptions about normality, as stated in Sections 4.1–4.3.

Extensions to a more general situation in which constant error variance is achieved using a Box–Cox transformed exposure scale are considerably more complex. No closed form expression for *E*(*X*_*i*_ | *W*_*i*1_,**Z**_*i*_) exists in general, and numerical integration methods are required to evaluate the expectation. There has not yet emerged a clear method for handling all types of heteroscedastic error using RC 40,41. However, some authors have found that ignoring heteroscedastic error and treating it as classical error may give rise to a good correction for error in many circumstances 40.

## 5. Correction methods allowing differential error

We have focussed so far on using RC to perform corrections for measurement error, and that is, to our knowledge, the most widely used approach. However, as noted earlier, RC relies on an assumption that any error is non-differential. In this section, we outline two more recently proposed approaches, MR and MI, which can accommodate differential error. Both methods explicitly allow for differential error because they aim to construct imputed values of the true exposure conditionally on all observed data, including the outcome. For a continuous exposure, Freedman *et al.* 7 found in simulation studies that MR and MI gave almost unbiased log OR estimates in a logistic regression using the continuous exposure.

We focus on the differential error model in 7, with error variances denoted 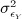 and error correlations *ρ*_*Y*_. We do not consider the use of these methods to additionally handle heteroscedastic error. The use of MI to correct for heteroscedastic error was investigated by Guo and Little 41, though they assumed a rather specific model for the heteroscedastic error and assumed that *X* was observed in some individuals, that is, they did not consider the repeated measures situation. To our knowledge, MR has not yet been considered for heteroscedastic error.

In the situation where we have repeated exposure measurements, parameters *ψ*_*Y*_, *θ*_*Y*_, and *ρ*_*Y*_ cannot be estimated, and sensitivity analyses would have to be used. In some settings, it may be reasonable to assume *ψ*_*Y*_ = 0, *θ*_*Y*_ = 1 and *ρ*_*Y*_ = 0, in which case no sensitivity analyses are required in the repeated measures situation.

To perform MR or MI allowing for differential error, repeated measures must be available for some individuals with *Y* = 0 and some with *Y* = 1. If MI and MR, as described in the succeeding text, are used when the error is in fact non-differential error, then these methods will in general result in a loss in efficiency relative to RC 7.

### 5.1. Moment reconstruction

Moment reconstruction was proposed by Freedman *et al.* 6 as a method for correcting for error in univariate continuous exposures. The idea of this approach is to find values *X*_*MR*_ such that the first two joint moments of *X*_*MR*_ with *Y* are the same as the first two joint moments of *X* with *Y*, that is, so that *E*(*X* | *Y* ) = *E*(*X*_*MR*_ | *Y* ) and var(*X* | *Y* ) = var(*X*_*MR*_ | *Y* ). The moment reconstructed value for individual *i*, *X*_*iMR*_, is obtained as a function of the mismeasured exposure *W*_*i*1_. It has been shown 6,7 that *X*_*iMR*_ can be obtained using the simple formula

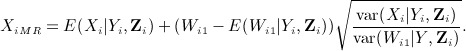
20 When the distributions of *X*_*i*_ and *W*_*i*1_ are jointly normal given *Y*_*i*_ and **Z**_*i*_, it follows the joint distribution of *X*_*iMR*_ and *Y*_*i*_ given **Z**_*i*_ is the same as that of *X*_*i*_ and *Y*_*i*_ given **Z**_*i*_ 6. This is in contrast to the RC values, which agree with the true exposure values only in expectation, but higher order moments will be different. The assumption of a joint normal distribution for *X*_*i*_ and *W*_*i*1_ given *Y*_*i*_ and **Z**_*i*_ is not required to obtain the moment reconstructed values in (20), and this approach has been found to work well under modest departures from that assumption 6.

The aforementioned MR formula allows both the expectation and variance to potentially depend on the outcome *Y*. Calculation of the MR values requires *E*(*X*_*i*_ | *Y*_*i*_,**Z**_*i*_) and var(*X*_*i*_ | *Y*_*i*_,**Z**_*i*_). Under an error model of the form in 7, these can be obtained using

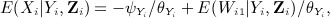
21

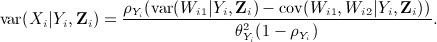
22 Note that in a validation study, these quantities can be estimated directly, provided *X*_*i*_ is observed for some individuals with *Y*_*i*_ = 1 and some with *Y*_*i*_ = 0.

The values *X*_*iMR*_ are used directly in place of *X*_*i*_ in the exposure-disease model, and it has been found that this results in unbiased estimates of linear exposure-disease associations 7. The parameters *ψ*_*Y*_, *θ*_*Y*_ and *ρ*_*Y*_ must be specified for each *Y* (*Y* = 0,1).

Thomas *et al.* 42 suggested an extension to the MR method proposed by Freedman *et al.* 6, called moment-adjusted imputation, which uses more than the first two moments to obtain imputed values for the true exposure, therefore allowing greater flexibility of the method, for example, for use with non-normally distributed exposures. MR has not, to our knowledge, been extended to a multivariate setting.

### 5.2. Multiple imputation

Multiple imputation was introduced by Rubin 43 and is becoming widely used to handle missing data in studies of different types. See White *et al.* 44,45 for recent summaries. Cole *et al.* 8 and Freedman *et al.* 7 proposed using MI to correct for measurement error in continuous exposures, by treating the true continuous exposure values as missing data. The key idea is that values of the true exposure are imputed by drawing a random value from the distribution of the true exposure conditional on all observed data, including the outcome. In the situation considered in this paper, the method therefore requires us to estimate the distribution of the true exposure *X* conditionally on *W*_1_, **Z** and *Y*, and also on *W*_2_ for individuals with a repeated exposure measurement. If we had a validation study within which *X* was observed, estimation of this distribution would follow procedures used in a more standard missing data setting. However, the situation that we focus on using repeated measures is non-standard. In both cases, to account for the uncertainty in the imputed values for the true exposure, a number of imputed values are obtained for each missing data point, creating *M* complete imputed data sets. We denote the *m*th imputed value for individual *i* by 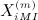. The exposure-outcome model is then fitted in each imputed data set, using 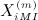 in place of *X*_*i*_, giving rise to *M* estimates of exposure-disease association, for example, the log OR in the case of logistic regression. The *M* estimates are combined to obtain a pooled estimate, using the so-called Rubin's Rules 43, which we outline in the succeeding text.

The true distribution of the true exposure *X* conditionally on *W*_1_, **Z** and *Y* is, in general, and in particular in the case of logistic regression models, a non-standard distribution. An approximate imputation model is therefore typically used. The most commonly used imputation model is of the form

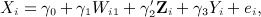
23 and for individual *i* the *m*th imputed true exposure is given by

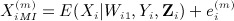


where 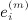 is a random draw from the distribution of the residuals from the regression of *X* on *W*_1_, **Z** and *Y*, that is from a normal distribution with mean 0 and variance var(*X* | *W*_1_,**Z**,*Y* ). To obtain the imputed values therefore requires the estimation of *E*(*X*_*i*_ | *W*_*i*1_,**Z**_*i*_,*Y*_*i*_) and var(*X*_*i*_ | *W*_*i*1_,**Z**_*i*_,*Y*_*i*_) using only the observed data. By assuming that *X* and *W*_1_ are jointly normally distributed given *Y* and **Z**, it can be shown, under the measurement error model in 5, that

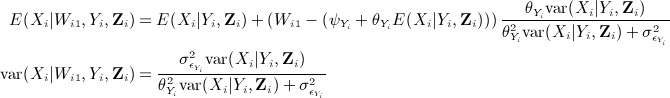
25 where the error variance 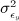 (*y* = 0,1) can be estimated by

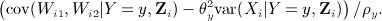


The terms *E*(*X*_*i*_ | *Y*_*i*_,**Z**_*i*_) and var(*X*_*i*_ | *Y*_*i*_,**Z**_*i*_) can be obtained as described in the previous section. As in MR, to perform MI therefore requires us to specify values for parameters *ψ*_*Y*_, *θ*_*Y*_ and *ρ*_*Y*_.

The exposure-outcome association estimates 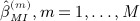 for each imputed dataset are combined to give a pooled estimate 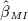, and corresponding variance, using the formulae

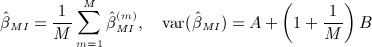
27 where *A* and *B* represent the within-imputation and between-imputation variance components, respectively. We have 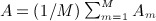, where *A*_*m*_ is the estimated variance of 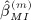, and 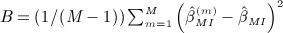. This variance estimate does not incorporate the variability due to estimation of the error model parameters and will tend to underestimate the true variance. Bootstrapping of the whole procedure of estimating the error model parameters and then *β* is probably the simplest approach to obtaining ‘correct’ standard errors.

For individuals with repeated exposure measurements, the imputation model in (23) can be extended to include a term in *W*_*i*2_, and imputed values are obtained using 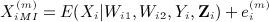, where 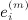 is a random draw from a normal distribution with mean 0 and variance var(*X*_*i*_ | *W*_*i*1_,*W*_*i*2_,*Y*_*i*_,**Z**_*i*_). By assuming {*X*_*i*_,*W*_*i*1_,*W*_*i*2_}are jointly normally distributed given *Y*_*i*_ and **Z**_*i*_, it can be shown that

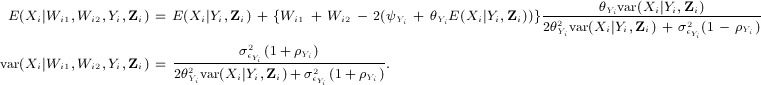
28

Multiple imputation extends to the multivariate setting by the use of chained equations, in which imputation models for each error-prone exposure conditional on all others, plus the other adjustment variables, are fitted iteratively 46,47, or by using an assumption such as a multivariate normal distribution for the full collection of true and observed exposures. If a validation study were available in which the true exposure *X* was observed, then standard software could be used to perform MI by chained equations. However, this is not possible in the situation where we have only repeated measures and extensions to the results in (25) and (28) would be required.

## 6. Correction methods for use when a continuous exposure is categorized

The focus so far has been on correction for the effects of error on parameters associated with the effects of a continuous exposure. In epidemiological analysis, exposures are commonly divided into categories, often quantiles, and the exposure-outcome association within each category is estimated relative to a reference category. We refer to this as a categorized exposure analysis. Although this approach has been criticized in the statistical literature 48, it is often used to assess possible non-linearity in the exposure-outcome model without having to specify a non-linear model for the continuous exposure. It also provides a convenient way of presenting results.

Measurement error in a continuous exposure can result in some individuals being misclassified when the exposure is categorized, resulting in biased estimates of relative associations between the exposure groups and disease risk. Flexible methods have not yet been developed for correcting for measurement error when continuous exposures are categorized. For a continuous exposure measured with error, individuals close to the category boundaries are more likely to be misclassified; hence, the classification according to the mismeasured exposure may provide additional information about disease risk beyond that in the categorized true exposure, and the misclassification error becomes dependent on the outcome. This means that even if error in the continuous exposure *W*_*ij*_ is non-differential, when it is categorized the misclassification will be differential 49.

Below we outline three methods for correcting for error in estimates associated with quantiles of a single dietary exposure. The methods implicitly assume the exposure-outcome association is linear: if not, they are biased towards linearity, but may nevertheless be useful in indicating strong departures from linearity.

### 6.1. A graphical method

MacMahon *et al*. 1 proposed a graphical method to obtain corrected parameter estimates within exposure categories from a categorized exposure analysis when the exposure is subject to classical measurement error and applied it in a study of diastolic blood pressure and risk of stroke and coronary heart disease. They noted that when using an exposure measured with error in a categorized exposure analysis, the lowest/highest category will include disproportionately many individuals whose single exposure measurement happened to be lower/higher than their ‘usual’ exposure. In the proposed correction method, the estimated OR (in the case of a logistic regression) within each exposure category is plotted against an unbiased estimate of the mean ‘usual’ exposure in that category. The means of repeated measurements within exposure categories defined by the original exposure measurements provide unbiased estimates of mean usual exposure within categories.

Although it has been fairly widely used, MacMahon's method does not work in general for non-linear associations; however, it has been found to perform well for quadratic associations 50. Also, this method assumes classical measurement error and does not account for multivariate measurement error.

### 6.2. Using imputation-based methods

Keogh *et al.* 50 recently investigated alternative approaches to correcting for the effects of measurement error in the categorized exposures situation. An attractive approach first obtains imputed values for the underlying continuous exposure *X*_*i*_ and then categorizes these in whatever way is of interest (fixed cutpoints or quantiles) for use in a categorized exposure analysis. The use of MR or MI to obtain imputed values of the true exposure, followed by categorization, has been found to work well for linear associations 50. Further investigations, not yet published, suggest that this approach may also extend to non-linear associations.

### 6.3. A method using regression calibration

We assume in this section that the exposure is to be categorized into quantiles and outline a method for estimating the association with the outcome of a 1-quantile increase in the dietary exposure. This provides an estimate of the trend in disease risk across quantiles of intake under the assumption of the trend being linear. We show in the succeeding text that this can provide a useful ‘trick’ for investigating the amount of correction required for measurement error in linear models.

We focus on a univariate exposure. For individual *i*, let 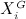 and 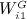 denote the true and observed quantiles of the continuous variables *X*_*i*_ and *W*_*i*1_, taking values in {1,2, … ,*Q*}, where *Q* is the number of quantiles. Treating 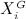 as a continuous variable, the exposure-outcome model of interest is now

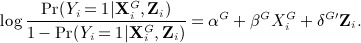
29 The corresponding naive model is

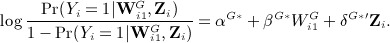
30 First, consider the case where the underlying continuous measurement *W*_*i*1_ follows the classical error model in 3. Under this error model, and assuming *X*_*i*_ and *W*_*i*1_ are normally distributed, it can be shown that *β*^*G*^ can be approximated by

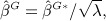
31 where, as before, *λ* is the RDR in model 9 and can be estimated by the regression coefficient for *W*_*i*1_ in a linear regression of *W*_*i*2_ on *W*_*i*1_ and **Z**_*i*_. Compare this with the result in (10), where the correction for a continuous exposure is 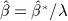. That is, the degree of correction for measurement error required under model (29) is smaller than that required under the continuous exposure-outcome model 1.

If the underlying continuous measurement *W*_*i*1_ has the systematic error model in 5, the result in (31) becomes

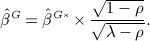
32

The results in (31) and (32) rely on the exposure categories being based on quantiles. It does not apply if the categories are based on fixed cutpoints. This method also holds if 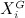 is replaced by the exposure on the standard deviation scale, that is, if the interest is in the effect of a standard deviation change in the exposure.

## 7. Illustration: fibre intake and colorectal cancer

We illustrate the methods outlined earlier using data from a matched case–control study of the association between fibre intake and colorectal cancer. The case–control study was sampled within the European Prospective Investigation into Cancer and Nutrition (EPIC)-Norfolk cohort, and the results have been presented elsewhere 9. Measures of dietary intake have been obtained for the case–control sample from a 7-day diary completed at least 12 months prior to diagnosis. Repeated 7-day diary measurements are available for a subset of participants. The repeated measurements were obtained from diaries collected approximately 4 years after the first diary was collected. The individuals for whom a repeated measure is available are not part of a random sample. The repeated measures have instead arisen in a fairly ad hoc way due to diaries being processed for other studies nested within the cohort. We use conditional logistic regression to estimate ORs for fibre intake in grams per day. The aforementioned methods extend to the setting of a conditional logistic regression provided the matching variables are included in **Z** when the correction procedures are performed. In this example, the matching is based on sex and age within 3 years, and we also adjust for non-dietary variables assumed to be measured without error: exact age, height, weight, social class, education level, smoking status and level of physical activity. The vector **Z** includes all adjustment variables and matching variables. For the purposes of this illustration, we restrict the analyses to 305 cases and 1222 controls with complete information for the adjustment variables, of whom 399 (26%) have a repeated diet diary measurement, although in practice, individuals with incomplete information should be included, for example, using MI.

**Figure 1:**
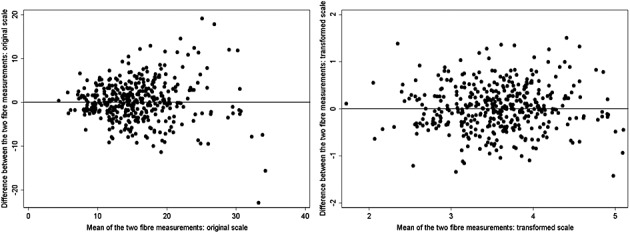
Results from a case–control study within the EPIC-Norfolk cohort. Plots of 7-day diary measurements of fibre intake on the original and log-transformed scales.

Figure 1 shows plots of the two fibre measurements on the original scale and on the log scale. By using the original scale measurements, there appears to be an increase in error variance as the measurements increase. On the log scale, the error variance appears constant. We therefore use the log-transformed measurements as the main exposure in our exposure-outcome analyses. In a secondary analysis, we consider the use of original scale measurements as the main exposure in our exposure-outcome analyses, while allowing for the error heterogeneity.

We focus here on a univariate dietary exposure as adjustment for energy intake has little impact on the estimates. We make the assumption of non-differential error because the food records were obtained prospectively.

All of the analyses were performed using R, and the code used is given in full in the Supporting Information.

### 7.1. Assuming classical measurement error

We first apply correction methods under the assumption of classical error on the log scale. The results from using RC, MR and MI are shown in Table 1, alongside the naive results. The error correction methods all give similar results. As we expect, the correction for error results in a stronger estimated association between fibre intake and colorectal cancer risk compared with the naive approach. The standard errors using MR and MI are larger than that found using RC. We expect this because MR and MI allow differential error, while RC assumes non-differential error.

**Table 1:** Results from a case–control study within the EPIC-Norfolk cohort; log odds ratio estimates (in units of 0.36 log scale grams per day, i.e. for an approximate 40% increase in fibre intake) from a naive analysis and using three methods for measurement error correction.

Method	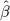	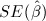 (a)	95% CI (a)	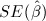 (b)	95% CI (b)
Naive analysis	− 0.193	0.070	( − 0.330, − 0.057)	NA	NA
Regression calibration	− 0.286	0.103	( − 0.488, − 0.085)	0.104	( − 0.490, − 0.082)
Moment reconstruction	− 0.311	0.088	( − 0.484, − 0.138)	0.145	( − 0.595, − 0.027)
Multiple imputation	− 0.313	0.108	( − 0.524, − 0.102)	0.140	( − 0.588, − 0.038)

We show standard errors (SE) and 95% confidence intervals (CI) (a) without allowing for the additional uncertainty in the measurement error estimation and (b) allowing for the additional uncertainty in the measurement error estimation. All methods were implemented using repeated measures in a subset of the study population.

**Figure 2:**
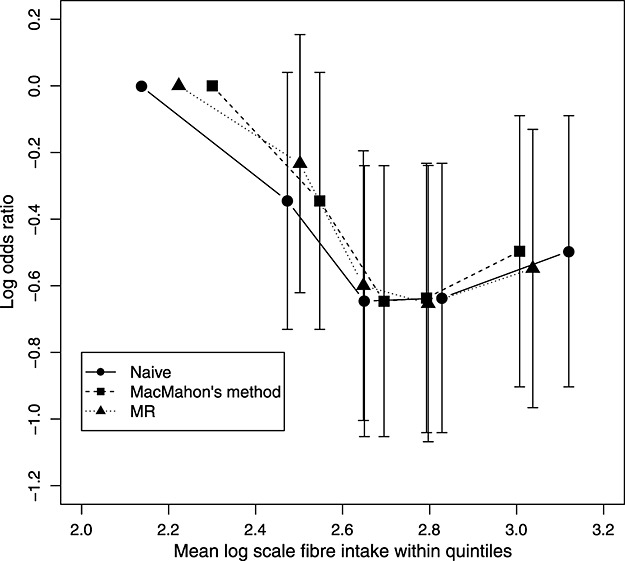
Results from a case–control study within the EPIC-Norfolk cohort. Log odds ratio estimates within quintiles of exposure: (i) naive method: naive log odds ratios within quintiles of observed log scale fibre intake plotted against mean observed exposure within quintile, (ii) MacMahon's method: naive log odds ratios are plotted against estimated usual intake within observed quintiles and (iii) moment reconstruction: log odds ratios within quintiles of true exposure (estimated using moment reconstruction) are plotted against mean estimated true exposure with those quintiles. Bars give 95% confidence intervals relative to the lowest quintile.

We also applied the correction methods for categorized exposures, described in Section 0018. Figure 2 shows the results from a naive analysis using quintiles of exposure, from MacMahon's method, and by imputing the underlying continuous exposure (on the log scale here) and categorizing that for use in the exposure-outcome analysis. In the uncorrected plot, there is some suggestion of a non-linear association. The results using the correction methods suggest a more non-linear relationship compared with the naive analysis, which is what we would expect 50.

### 7.2. Using sensitivity analyses to assess impact of systematic error

In this section, we extend the aforementioned results to incorporate sensitivity analyses to assess the potential impact of systematic errors, as under the error model in 5. We take plausible values for the sensitivity analyses from a study within a subset of the EPIC-Norfolk cohort described by Day *et al.* 15 in which they estimated measurement error models for 7-day diet diary measurements of protein, potassium and sodium using objective biomarkers for each nutrient from 24-h urine samples, enabling estimation of *θ* and *ρ* in 5. For the nutrients protein, potassium and sodium, the estimates of *θ* were, respectively, 0.81, 0.69 and 0.47, and the estimates of error correlations *ρ* were, respectively, 0.52, 0.58 and 0.52. The low estimate of *θ* = 0.47 for sodium is not unexpected because it is known that sodium intake is difficult to measure using this instrument. The higher estimates for *θ* for protein and potassium may therefore be more transferable to other nutrients. A small number of other studies have made similar estimates of systematic errors in 24-h recall measurements 18,17,16

In our sensitivity analyses, we considered values for *θ* of 1, 0.75 and 0.5 and values for *ρ* of 0 and 0.5. The results are shown in Table 2. As the value of *θ* moves away from 1, the strength of the error-corrected association is reduced, that is, it moves closer to the null. Correlation between the errors in the repeated measurements results in estimates showing a strengthening of the association, that is, a move away from the null. The significance of the association is approximately unchanged in these analyses.

**Table 2:** Results from a case–control study within the EPIC-Norfolk cohort; regression calibration with sensitivity analyses: log odds ratio estimates (95% CI) for log scale fibre intake (in units of 0.36 log scale grams per day) for different values of *θ* and *ρ*.

	*ρ* = 0	*ρ* = 0.5
*θ* = 1	− 0.286 ( − 0.490, − 0.082)	− 0.549 ( − 1.000, − 0.097)
*θ* = 0.75	− 0.215 ( − 0.368, − 0.062)	− 0.411 ( − 0.750, − 0.073)
*θ* = 0.5	− 0.143 ( − 0.245, − 0.041)	− 0.274 ( − 0.500, − 0.049)

Confidence intervals were obtained using corrected standard errors.

### 7.3. Allowing for heteroscedastic error

In the preceding text, we assumed a linear association between the log-transformed exposure and the outcome on a logistic scale. This was because the log scale measurements were approximately normally distributed and displayed approximately constant error variance (Figure 1). Suppose instead that interest was in an assumed linear association between the untransformed exposure and the outcome on a logistic scale. In this situation, we apply the special RC methods outlined for this situation in Section 4.4. We also apply standard RC, assuming classical error in the untransformed measurements, even though this appears not to be the case. The results are shown in Table 3. The results from the two RC approaches are similar, suggesting that in this case assuming classical error to perform the RC was reasonable.

**Table 3:** Results from a case–control study within the EPIC-Norfolk cohort; log odds ratios for a 6 grams per day increase in fibre intake estimated using a naive analysis, using regression calibration assuming classical error in the untransformed measurements and using regression calibration allowing for the heteroscedastic error.

Method	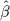	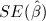 (a)	95% CI (a)	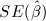 (b)	95% CI (b)
Naive	− 0.200	0.081	( − 0.360, − 0.041)	NA	NA
RC—assuming classical error	− 0.309	0.125	( − 0.555, − 0.063)	− 0.127	( − 0.558, − 0.061)
RC—heteroscedastic approach	− 0.319	0.124	( − 0.561, − 0.076)	0.153	( − 0.620, − 0.018)

We show standard errors (SE) and 95% confidence intervals (CI) (a) without allowing for the additional uncertainty in the measurement error estimation and (b) allowing for the additional uncertainty in the measurement error estimation. In (b), the standard errors were obtained using Equation (12) for regression calibration (RC) assuming classical error and using bootstrapping for the heteroscedastic approach.

The scale on which the exposure is used in the exposure-outcome model is a subject matter choice. In this example, either the original scale or the log scale may be reasonable. Figure 2 gives little evidence to choose between a linear association on the transformed or untransformed scale.

## 8. Discussion

The aim of this paper has been to provide in one place a toolkit of methods for correcting for the effects of exposure measurement error on exposure-outcome associations. The focus was on making such corrections by utilizing repeated measures of the error-prone exposure. We were motivated by the need for such correction methods in nutritional epidemiology, where some studies are now using repeated food records to measure dietary intake. Food records have often been assumed to be subject only to random errors, but there is good evidence to the contrary, and one aspect of this paper was to outline methods for sensitivity analysis to assess the impact of systematic errors. Again, our methods apply equally for FFQ measurements. The broader aim was to cover a range of types of error and methods, which are relevant in a broader context.

Table 4 provides an overview of the methods considered and the measurement error settings in which they may be used, based on established developments to date. It is probable that MI and MR can be extended for use in a wider range of circumstances in future work.

**Table 4:** Summary of when different error correction methods are appropriate for use.

Correction method	Classical error (Model 3)	Systematic error (Model 5)	Heteroscedastic error (Model 6)	Differential error (Model 7)	Notes
Single error-prone exposure RC ( Section 0010)	*✓*	*✓*Given *θ*,*ρ*	*✓*If constant error variance after a log transformation	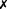	For heteroscedastic error: extensions to Box–Cox transformations are possible but require numerical integration. The standard RC approach may work well even when there is heteroscedastic error.
MR (Section 5.1)	*✓*	*✓*Given *θ*,*ρ*	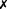	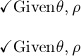	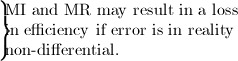
MI (Section 5.2)	*✓*	*✓*Given *θ*,*ρ*	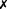		
Multiple error-prone exposures					
RC ( Section 0010)	*✓*	*✓*Given *θ*^(*k*)^,*ρ*^(*k*)^,	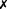	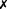	Many parameters need to be specified to handle systematic error.
		(*k* = 1, … ,*K*)			
					
MI (Section 5.2)	*✓*	*✓*Given *θ*^(*k*)^,*ρ*^(*k*)^,	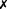	*✓*	MI using chained equations could be used when there is a validation sample. Extensions to the repeated measures setting are feasible, for example, using joint normality assumptions, but could not be performed easily in standard software.
		(*k* = 1, … ,*K*)			
Single error-prone exposures categorized in the exposure-outcome model					
Macmahon's method (Section 6.1)	*✓*	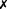	*✓*	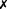	This method typically underestimates non-linearity.
MR (Section 6.2)	*✓*	*✓*Given *θ*,*ρ*	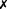	*✓*	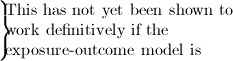 non-linear. Adaptations may be needed in this case.
MI (Section 6.2)	*✓*	*✓*Given *θ*,*ρ*	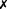	*✓*	
RC (Section 6.3)	*✓*	*✓*Only if a linear trend is assumed across categories, given *ρ*	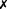	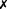	RC can be used under classical or systematic error (given *ρ*) only if a linear trend is assumed across categories.

RC, regression calibration; MR, moment reconstruction; MI, multiple imputation.

We emphasized the use of RC to make corrections for measurement error, this being the most popular approach in practice. We also showed how RC can be extended to incorporate sensitivity analyses to investigate the potential impact of departures from classical error on corrected estimates of exposure-outcome associations. The use of sensitivity analyses such as these is important in nutritional epidemiology, where there is evidence of systematic errors in dietary measurements, but few unbiased measures with which to make comparisons. If a validation study is available, in which the true exposure is observed, then no sensitivity analyses are necessary.

Regression calibration does not apply when exposure measurement error is differential. We summarized two correction methods for use in this situation: MR and MI. Both are relatively new, in the context of measurement error correction, and have not yet to our knowledge been used extensively. Both methods are reasonably straightforward to implement when repeated error-prone exposure measurements are available, and even more so if the true exposure can be observed in a validation study. If it is considered appropriate to assume non-differential error, then RC is likely to be the most efficient approach. Depending on the form of the differential error, if only repeated exposure measurements are available, then some parameters may have to be specified and used in sensitivity analyses. If there is a validation study, then the form of the differential error can be estimated, and no sensitivity analyses are required, provided the validation sample contains both cases and controls.

Heteroscedastic measurement error is likely to occur quite commonly. If a transformation can be made under which the errors have constant variance, then exposure-outcome associations can be studied using the transformed exposure, applying any correction methods on the transformed scale. However, this may not always be appropriate. Some approaches have been recently suggested for correcting for heteroscedastic error when it is desirable to use the untransformed exposure in the main analysis 41,40, but a general method is still lacking. Note that if there is a validation study, the form of the heteroscedastic error could be investigated directly; however, this is not possible when we must use repeated exposure measurements, where more general methods are required to deal with non-constant error variance. It is possible that MR and MI could be extended for use with this type of error, and this is an area for further work. It is also quite possible that RC assuming classical error works adequately in many circumstances where the error is in fact heteroscedastic 40.

We also presented three approaches to correcting for error in estimates obtained in an analysis based on a categorization of the continuous exposure. When the exposure-outcome association is linear, MacMahon's method for categorized exposures gives a way of showing graphically the corrected association. However, this method assumes linearity and, moreover, gives purely graphical results. Development of measurement error correction methods for general use in grouped exposure analyses based on categorized continuous exposures is an important area for future work. No method has yet been developed for categorized exposures, which allows for underlying non-linearity in the exposure-disease association or non-classical measurement error. Instead, we outlined a method for investigating the degree of measurement error correction required under an assumption of a linear association across quantiles. As in any setting, categorization of exposures should be used with caution.

There are many methods for error correction that we have not covered in this paper. These include full likelihood approaches 4 and simulation extrapolation 51. Although we considered categorized exposure methods, we did not consider methods for correcting for measurement error under non-linear models for the exposure-outcome association. RC has been extended to the non-linear situation. A simple method for investigating non-linearity in exposure-outcome associations is to include a quadratic term for the main exposure in the exposure-outcome model. By using RC, the parameters associated with linear and quadratic terms are estimated by replacing *X* and *X*^2^ with *E*(*X* | *W*_1_,**Z**) and *E*(*X*^2^ | *W*_1_,**Z**). The second term can be computed by assuming a particular distribution for *X* | *W*_1_,**Z**. More flexible methods for investigating non-linearity include modelling the exposure using penalized splines or fractional polynomials. The idea of RC has been developed for use with splines 52, as has simulation extrapolation 52,53. One issue specifically relevant to the use of repeated food records in nutritional epidemiology is the potential for zero-inflated measurements, which can occur when some individuals do not report eating the food in question during the period of the food record. Special models have been developed for use in this situation 54,55. Some of the other error correction methods described in the succeeding text, in particular those for use in non-linear models, are complex, and software for implementing them has not yet become available. For this reason, these methods have not been widely used in practical applications in epidemiology.

In summary, we have presented a collection of methods for error correction, which can be applied to deal with error of different types when repeated error-prone exposure measurements are available. We have provided guidance for practical application of the methods. All of the methods we described can be applied using familiar methods in standard software, and an example code using R software is given in the Supporting Information. Their application involves calculation of expectations and variances and using linear regression models.

## Supporting Information

Supporting info item
