# Supplementary material for: A toolkit for measurement error correction, with a focus on nutritional epidemiology
Source: Stat Med. 2014 Feb 4;33(12):2137–55. doi: 10.1002/sim.6095 (PMC4285313; doi:10.1002/sim.6095)
Supplement: Supplementary file 1 — Supporting info item [file sim0033-2137-sd1.pdf]

A toolkit for measurement error correction, with a focus on nutritional epidemiology: Supplementary materials

Ruth H. Keogh, Ian R. White

In this document we provide the R code used to perform the correction methods used in the illustrative example, as described in Section 7. We use generic labels for the variables and the code could easily be adapted for other studies. The data is from a matched case-control study of fibre intake and colorectal cancer and conditional logistic regression is used. We use the following notation:

- The case-control data is called `mydata`.
- The outcome variable `y` takes value 1 for cases and 0 for controls.
- The main exposure is fibre intake measured using a diet diary. The original scale measurement is labelled `w1` and the log scale measurement is `w1.log`. Repeated measures are available for a subset of individuals, and the corresponding measures are labelled `w2` and `w2.log` (recorded as `"NA"` for individuals without a repeated measure).
- The matching variables are age group and sex and we denote the vector of matching variables by `z.m`. Additional variables to be adjusted for are exact age, height, weight, smoking status, social class, physical activity level, and education level, of which all but height and weight and exact age are categorical variables. The vector of adjustment variables is denoted `z`. Note that it is sufficient in this example for `z.m` to contain only the information on sex because exact age appears as an adjustment variable.
- Matched sets are identified by the variable `match`.

Naive analysis (Table 1)

The naive analysis is performed as follows:

```
library(survival)

naive.analysis<-clogit(y~w1.log+z+strata(match),data=mydata)
```

Regression calibration (RC) (Table 1)

RC for classical measurement error was outlined in Section 4.1. To perform RC we first fit the RC model, which is a regression of the second exposure measurement on the first and on all adjustment variables including the matching variables:

```
rc.model<-lm(w2.log~w1.log+z+z.m,data=mydata)
rc.fitted<-predict.lm(rc.model,mydata)
```

The expectations $E(X|W_1, \mathbf{Z})$ are given by `rc.fitted`, and the corrected odds ratio estimates are found by using this as the main exposure in the analysis model:

```
rc.analysis<-clogit(y~rc.fitted+z+strata(match),data=mydata)
```

The variance of the corrected log odds ratio estimate (given by the coefficient of `rc.fitted` in the above model) is underestimated in the above model because it does not take into account the uncertainty in the parameters estimated in the regression calibration model. The corrected variance estimate (equation (12)) can be obtained as follows:

```
var.betastar<-naive$var[1,1]
lambda<-rc.model$coef[2]
betastar<-naive$coef[1]
var.lambda<-vcov(rc.model)[2,2]

var.corrected<-(var.betastar/(lambda^2))+((betastar/(lambda^2))^2)*var.lambda
```

Regression calibration can be performed in Stata using the `rcal` command in the `merror` package (http://www.stata.com/merror/), which accommodates the situation using repeated measures and gives bootstrap estimates for the variance of the corrected estimate. It does not incorporate sensitivity analyses to allow for systematic error.

Moment reconstruction (MR) (Table 1)

To use MR we first need to estimate $E(X|Y,\mathbf{Z})$, var$(X|Y,\mathbf{Z})$. For $Y=1$ these are given respectively below by `exp.x.y1z` and `var.x.y1z`, and for $Y=0$ by `exp.x.y0z` and `var.x.y0z`. Following the results given in Section 5.1, the calculations are performed as follows:

```
mydata2<-subset(mydata,mydata$w2.log!="NA")

mr.model.y1<-lm(w1.log~z+z.m,data=mydata[mydata$y==1,])
mr.model1b.y1<-lm(w1.log~z+z.m,data=mydata2[mydata2$y==1,])
mr.model2b.y1<-lm(w2.log~z+z.m,data=mydata2[mydata2$y==1,])
var.w1.y1z<-var(mr.model.y1$res)
exp.x.y1z<-predict(mr.model.y1,mydata)
var.x.y1z<-cov(mr.model1b.y1$res,mr.model2b.y1$res)

mr.model.y0<-lm(w1.log~z+z.m,data=mydata[mydata$y==0,])
mr.model1b.y0<-lm(w1.log~z+z.m,data=mydata2[mydata2$y==0,])
mr.model2b.y0<-lm(w2.log~z+z.m,data=mydata2[mydata2$y==0,])
var.w1.y0z<-var(mr.model.y0$res)
exp.x.y0z<-predict(mr.model.y0,mydata)
var.x.y0z<-cov(mr.model1b.y0$res,mr.model2b.y0$res)
```

Using the above, the moment reconstructed values are given by

```
x.mr.y1<-exp.x.y1z+(mydata$w1.log-exp.x.y1z)*sqrt(var.x.y1z/var.w1.y1z)
x.mr.y0<-exp.x.y0z+(mydata$w1.log-exp.x.y0z)*sqrt(var.x.y0z/var.w1.y0z)

x.mr<-ifelse(mydata$y==1,x.mr.y1,x.mr.y0)
```

Finally, the analysis model is fitted using the moment reconstructed values as the main exposure:

```
mr.analysis<-clogit(y~x.mr+z+strata(match),data=mydata)
```

The standard error and 95% confidence interval which do now allow for uncertainty in the measurement error estimation ((a) in Table 1) arise directly from the analysis model just fitted. To account for the error in estimation of the measurement error we used bootstrapping ((b) in Table 1). Because this is a matched case-control study we sampled matched sets rather than individuals (note that there are a total of 318 matched sets). We obtained 1000 bootstrap samples, performed MR within each sample, and fitted the analysis model using the moment reconstructed values. The standard error of interest is given by the standard deviation of the 1000 bootstrap estimates of the log odds ratio β (`est.boot`). We used the code given below:

```
n.boot<-1000
est.boot<-rep(0,n.boot)
```

```
for(j in 1:n.boot){
boot.sample<-sample(levels(mydata$match),size=318,replace=T)

row.no.new<-NULL
for(i in 1:length(names(table(boot.sample)))){
row.no<-rep(which(mydata$match %in% names(table(boot.sample))[i]),
table(boot.sample)[i])
row.no.new<-c(row.no.new,row.no)
}

mydata.boot<-mydata[row.no.new,]

mydata2.boot<-subset(mydata.boot,mydata.boot$w2.log!="NA")
mr.model.y1<-lm(w1.log~z+z.m,data=mydata.boot[mydata.boot$y==1,])
mr.model1b.y1<-lm(w1.log~z+z.m,data=mydata2.boot[mydata2.boot$y==1,])
mr.model2b.y1<-lm(w2.log~z+z.m,data=mydata2.boot[mydata2.boot$y==1,])
var.w1.y1z<-var(mr.model.y1$res)
exp.x.y1z<-predict(mr.model.y1,mydata.boot)
var.x.y1z<-cov(mr.model1b.y1$res,mr.model2b.y1$res)

mr.model.y0<-lm(w1.log~z+z.m,data=mydata.boot[mydata.boot$y==0,])
mr.model1b.y0<-lm(w1.log~z+z.m,data=mydata2.boot[mydata2.boot$y==0,])
mr.model2b.y0<-lm(w2.log~z+z.m,data=mydata2.boot[mydata2.boot$y==0,])
exp.x.y0z<-predict(mr.model.y0,mydata.boot)
var.w1.y0z<-var(mr.model.y0$res)
var.x.y0z<-cov(mr.model1b.y0$res,mr.model2b.y0$res)

x.mr.y1<-exp.x.y1z+(mydata.boot$w1.log-exp.x.y1z)*sqrt(var.x.y1z/var.w1.y1z)
x.mr.y0<-exp.x.y0z+(mydata.boot$w1.log-exp.x.y0z)*sqrt(var.x.y0z/var.w1.y0z)

x.mr<-ifelse(mydata.boot$y==1,x.mr.y1,x.mr.y0)

mr.analysis<-clogit(y~x.mr+z+strata(match),data=mydata.boot)
est.boot[j]<-mr.analysis$coef[1]
}
```

Multiple imputation (MI) (Table 1)

To use MI we first need to estimate the expectation $E(X|W_1,Y,\mathbf{Z})$ and variance var$(X|W_1,Y,\mathbf{Z})$, as outlined in Section 5.2. These estimates were obtained as follows:

```
mr.model.y1<-lm(w1.log~z+z.m,data=mydata[mydata$y==1,])
exp.w1.y1z<-predict(mr.model.y1,mydata)
exp.x.w1y1z<-(mydata$w1.log*cov.w1w2.y1z+exp.w1.y1z*(var.w1.y1z-cov.w1w2.y1z))/var.w1.y1z
var.x.w1y1z<-(var.w1.y1z-cov.w1w2.y1z)*cov.w1w2.y1z/var.w1.y1z

mr.model.y0<-lm(w1.log~z+z.m,data=mydata[mydata$y==0,])
exp.w1.y0z<-predict(mr.model.y0,mydata)
exp.x.w1y0z<-(mydata$w1.log*cov.w1w2.y0z+exp.w1.y0z*(var.w1.y0z-cov.w1w2.y0z))/var.w1.y0z
var.x.w1y0z<-(var.w1.y0z-cov.w1w2.y0z)*cov.w1w2.y0z/var.w1.y0z

exp.x.w1yz<-ifelse(mydata$y==1,exp.x.w1y1z,exp.x.w1y0z)
var.x.w1yz<-ifelse(mydata$y==1,var.x.w1y1z,var.x.w1y0z)
```

We obtained 10 imputed values for the true exposure and fitted the analysis model in each case as follows:

```
library(mice)
m<-10
n<-length(mydata$rkid)
```

```
mi.coef<-rep(0,m)
mi.var<-rep(0,m)
for(i in 1:m){
x.mi<-rnorm(n,exp.x.w1yz,sqrt(var.x.w1yz))
mi.coef[i]<-clogit(y~x.mi+z+strata(match),data=mydata)$coef[1]
mi.var[i]<-clogit(y~x.mi+z+strata(match),data=mydata)$var[1,1]
}
mi.pool<-pool.scalar(mi.coef,mi.var)
```

The pooled hazard ratio estimate for the main exposure is given by `mi.pool$qbar`, and its variance by `mi.pool$qbar`. Note that `pool.scalar` is part of the `mice` package.

We used a bootstrapping procedure the same as that outlined for MR in the above section. The details are not repeated here.

In the situation in which the true exposure X is observed in a validation sample, MI can be performed in R using the `mice` package [1], for example, and in Stata using the `ice` package [2].

Regression calibration with sensitivity analyses (Table 2)

Use of RC for systematic error was discussed in Section 4.3. In the example, sensitivity analyses were used to assess the impact of different values for parameters θ, ρ. We show the code used to perform the analysis for one particular set of sensitivity parameter values, where the corrected odds ratio estimate is given by `beta.corrected` and the corrected variance by `var.corrected`:

```
rc.model<-lm(w2.log~w1.log+z+z.m,data=mydata)

rho<-0.75
theta<-0.5

lambda<-(rc.model$coef[2]-rho)/(beta*(1-rho))
betastar<-naive$coef[1]

corrected.beta<-betastar/lambda

var.betastar<-naive$var[1,1]

var.lambda<-vcov(rc.model)[2,2]/((theta^2)*((1-rho)^2))

var.corrected.beta<-(var.betastar/(lambda^2))+((betastar/(lambda^2))^2)*var.lambda
```

Methods for categorized exposures (Figure 2)

For the categorized exposures analyses described in Section 6, assuming classical error, we divided the main exposure into quintiles. The naive categorised exposure analysis, including the results plot (see Figure 2), can be performed as follows:

```
w1.q<-cut(mydata$w1.log,breaks=quantile(mydata$w1.log,probs=seq(0,1,0.2)),
labels=F,include.lowest=T)

naive.q<-clogit(y~as.factor(w1.q)+z+strata(match),data=mydata)

mean.q<-c(mean(mydata$w1.log[w1.q==1]),mean(mydata$w1.log[w1.q==2]),
mean(mydata$w1.log[w1.q==3]),
mean(mydata$w1.log[w1.q==4]),mean(mydata$w1.log[w1.q==5]))

ci.lower<-naive.q$coef[1:4]-1.96*sqrt(diag(naive.q$var[1:4,1:4]))
ci.upper<-naive.q$coef[1:4]+1.96*sqrt(diag(naive.q$var[1:4,1:4]))
```

```
plot(mean.q,c(0,naive.q$coef[1:4]),pch=16,cex=1.5,ylab="Log odds ratio",
xlab="Mean log scale fibre intake within quintiles",xlim=c(2,3.2),
ylim=c(-1.2,0.2),type="b")
arrows(x0 = mean.q, x1 = mean.q, y0 = c(0,ci.lower), y1 = c(0,ci.upper),
 length=0.05,angle=90,code=3,col=1)
```

MacMahon's method was performed as follows:

```
mean.q.mm<-c(mean(mydata$w2.log[w1.q==1],na.rm=T),
mean(mydata$w2.log[fibre.q==2],na.rm=T),
mean(mydata$w2.log[fibre.q==3],na.rm=T),
mean(mydata$w2.log[fibre.q==4],na.rm=T),
mean(mydata$w2.log[fibre.q==5],na.rm=T))

points(mean.q.mm,c(0,naive.q$coef[1:4]),pch=15,cex=1.5,type="b",lty=2)
arrows(x0 = mean.q.mm, x1 = mean.q.mm, y0 = c(0,ci.lower), y1 = c(0,ci.upper),
length=0.05,angle=90,code=3,col=1)
```

Finally, we performed the categorized exposures analysis using the moment reconstructed values. These were calculated above (`x.mr`) and the categorized analysis was performed as follows:

```
w1.q.mr<-cut(x.mr,breaks=quantile(x.mr,probs=seq(0,1,0.2)),
labels=F,include.lowest=T)
mean.q.mr<-c(mean(x.mr[w1.q.mr==1],na.rm=T),
mean(x.mr[w1.q.mr==2],na.rm=T),mean(x.mr[w1.q.mr==3],na.rm=T),
mean(x.mr[w1.q.mr==4],na.rm=T),mean(x.mr[w1.q.mr==5],na.rm=T))

mr.q<-clogit(casecolo~as.factor(w1.q.mr)+z+strata(match),data=mydata)
ci.lower<-mr.q$coef[1:4]-1.96*sqrt(diag(mr.q$var[1:4,1:4]))
ci.upper<-mr.q$coef[1:4]+1.96*sqrt(diag(mr.q$var[1:4,1:4]))

points(mean.q.mr,c(0,mr.q$coef[1:4]),pch=17,cex=1.5,type="b",lty=3)
arrows(x0 = mean.q.mr, x1 = mean.q.mr, y0 = c(0,ci.lower), y1 = c(0,ci.upper),
length=0.05,angle=90,code=3,col=1)

legend(2,-0.8,pch=c(16,15,17),lty=c(1,2,3),legend=c("Naive","MacMahon's method","MR"))
```

Allowing heteroscedastic error (Table 3)

Finally, we applied the methods for heteroscedastic error correction described in Section 4.4 to the example data (Section 7.3). Here it is assumed that fibre intake on the *original scale* is the exposure of interest.

If we ignore the evidence for heteroscedastic error on the original scale, then RC can be performed as follows:

```
rc.model<-lm(w1~w2+z+z.m,data=mydata)
rc.fitted<-predict.lm(rc.model,mydata)
rc.analysis<-clogit(y~rc.fitted+z+strata(match),data=mydata)
```

The standard error for β allowing for the uncertainty in the measurement error estimation was obtained using the approximation illustrated previously and the details are not given here.

The alternative method, in which we assume constant error variance on the log transformed scale was performed as follows:

```
mr.model<-lm(w1.log~z+z.m,data=mydata)
mydata2<-subset(mydata,mydata$w2.log!="NA")
mr.model1b<-lm(w1.log~z+z.m,data=mydata2)
mr.model2b<-lm(w2.log~z+z.m,data=mydata2)

mean.w.z<-predict(mr.model,mydata)
sigsq.u<-var(mr.model$res)-cov(mr.model1b$res,mr.model2b$res)
sigsq.x<-cov(mr.model1b$res,mr.model2b$res)
```

```
sigsq.w<-var(mr.model$res)
exp.x.w1z<-exp(0.5*sigsq.u+((mydata$w1.log*sigsq.x+mean.w.z*sigsq.u)/sigsq.w)+
            0.5*sigsq.x*sigsq.u/sigsq.w)

rc.hetero.analysis<-clogit(y~exp.x.w1z+z+strata(match),data=mydata)
```

Bootstrapping of this full procedure was used to obtain corrected standard error. The bootstrapping procedure was shown above and we do not give the details again here.

References

[1] Van Buuren S, Groothuis-Oudshoorn K. mice: Multivariate imputation by chained equations in R. *Journal of Statistical Software* 2011; **45**:1–67.

[2] Royston P, White I. Multiple Imputation by Chained Equations (MICE): Implementation in Stata. *Journal of Statistical Software* 2011; **45**:1–20.
